# Visual resemblance and interaction history jointly constrain pictorial meaning

**DOI:** 10.1038/s41467-023-37737-w

**Published:** 2023-04-17

**Authors:** Robert D. Hawkins, Megumi Sano, Noah D. Goodman, Judith E. Fan

**Affiliations:** 1 Department of Psychology, Stanford University, Stanford, CA USA; 2 Department of Psychology, Princeton University, Princeton, NJ USA; 3 Department of Computer Science, Stanford University, Stanford, CA USA; 4 Department of Psychology, University of California, San Diego, SC USA

**Keywords:** Human behaviour, Social behaviour

## Abstract

How do drawings—ranging from detailed illustrations to schematic diagrams—reliably convey meaning? Do viewers understand drawings based on how strongly they resemble an entity (i.e., as images) or based on socially mediated conventions (i.e., as symbols)? Here we evaluate a cognitive account of pictorial meaning in which visual and social information jointly support visual communication. Pairs of participants used drawings to repeatedly communicate the identity of a target object among multiple distractor objects. We manipulated social cues across three experiments and a full replication, finding that participants developed object-specific and interaction-specific strategies for communicating more efficiently over time, beyond what task practice or a resemblance-based account alone could explain. Leveraging model-based image analyses and crowdsourced annotations, we further determined that drawings did not drift toward “arbitrariness,” as predicted by a pure convention-based account, but preserved visually diagnostic features. Taken together, these findings advance psychological theories of how successful graphical conventions emerge.

## Abstract (web summary)

Drawings can vary in abstraction while still being meaningful. Here, the authors leverage a two-player drawing game to evaluate a cognitive account of pictorial meaning in which both visual and social information jointly support visual communication.

## Introduction

Human communication goes well beyond the exchange of words. Throughout human history, people have devised a variety of technologies to externalize and share their ideas in more durable visual formats. Perhaps the most basic and versatile of these technologies is drawing, which predates the invention of writing^1–3^ and is pervasive across many cultures^4^. The expressiveness of drawings has long provided inspiration for scientists investigating the mental representation of concepts in children^5,6^ and clinical populations^7,8^. Yet current theories of depiction fall short of explaining how humans are capable of leveraging drawings in such varied ways. In particular, it is not clear how drawing enables the flexible expression of meanings across different levels of visual abstraction, ranging from realistic depictions to schematic diagrams. Do viewers understand drawings based solely on their ability to resemble the entities they refer to (i.e., as images), or do they understand drawings based on shared but arbitrary associations with these entities (i.e., as symbols)?

On the one hand, there is strong evidence in favor of the image-based account, insofar as general-purpose visual processing mechanisms are sufficient to explain how people are able to understand what drawings mean. Recent work has shown that features learned by deep convolutional neural network models (DCNNs) trained only to recognize objects in photos, but have never seen a line drawing, nevertheless succeed in recognizing simple drawings^9^. These results provide support for the notion that perceiving the correspondence between drawings and real-world objects can arise from the same general-purpose neural architecture evolved to handle natural visual inputs^10–12^, rather than relying on any special mechanisms dedicated to handling drawn images. Further, visually evoked representations of an object in human visual cortex measured with fMRI can be leveraged to decode the identity of that object during drawing production, suggesting that functionally similar neural representations are recruited during both object perception and drawing production^13^. Together, these findings are convergent with evidence from comparative, developmental, and cross-cultural studies of drawing perception. For example, higher non-human primates^14^, human infants^15^, and human adults living in remote regions without pictorial art traditions and without substantial contact with Western visual media^16^ are all able to recognize line drawings of familiar objects, even without substantial prior experience with drawings.

On the other hand, other work has supported a symbol-based account, by pointing out the critical role that conventions play in determining how drawings denote objects^17,18^. What characterizes such conventional accounts is that they rely on associative learning mechanisms that operate over socially mediated experiences, beyond pre-existing perceptual competence. This view is supported by developmental^19^ and computational modeling^20^ work that has highlighted the importance of social context for explaining how people can robustly identify the referent of even very sparse drawings. Moreover, several pioneering experimental studies have identified a key role for real-time social feedback during visual communication in driving the increased simplification of drawings over time^21,22^, broadly consistent with the possibility that similar pressures shaped the emergence of modern symbol systems^23–25^. Further support for the notion that the link between pictures and their referents depends crucially on socially mediated learning comes from the substantial variation in pictorial art traditions across cultures^4^ and the existence of culturally specific strategies for encoding meaning in pictorial form^26–28^.

In this paper, we evaluate a cognitive account of pictorial meaning that aims to reconcile these resemblance-based and convention-based perspectives. According to our account, people integrate information from current visual experience with previously learned associations to determine the meaning of a drawing. Our account aims to ground foundational ideas from semiotic theory in specific cognitive processes that are integrated in a model of the viewer. Most notably, while C.S. Peirce’s taxonomy of signs^29^ is often glossed as making categorical distinctions between icons, indexes, and symbols, it is also compatible with a more continuous conceptualization of how different signs relate to one another^30–32^, where the meaning of a sign generally depends on interactions between "Firstness" (i.e., natural resemblance) and "Thirdness" (i.e., conventionalized relations). Similar arguments have also been advanced more recently in the philosophy literature on depiction, which continues to debate the merits of and objections to resemblance-based and convention-based views ^33,34,35^.

Our account makes two key predictions: First, while visual resemblance tends to dominate in the absence of learned associations, novel associations can emerge quickly and come to strongly determine pictorial meaning. For example, as two communicators learn to more strongly associate a particular drawing with an object it is intended to depict, even sparser versions of that drawing that share key visual features should still successfully evoke the original object, even if it directly resembles that object to a lesser extent. Second, visual resemblance will constrain the kinds of novel associations that form, such that visual information that is inherently more diagnostic of the referent will be more likely to form the basis for ad hoc graphical conventions. For example, if a target object is distinguished by a particular visual attribute (e.g., a particularly long beak for a bird), then it is more likely that the sparser drawing will preserve this attribute, even at the expense of other salient attributes of the target object.

**Fig. 1:**
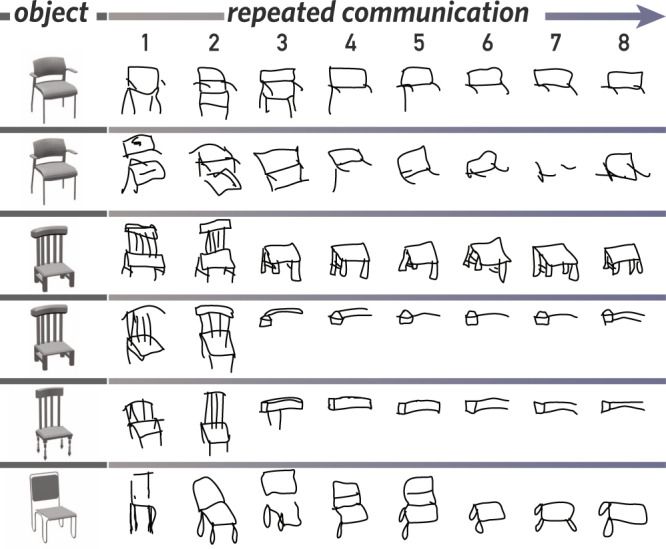
Examples of sketches produced in a repeated visual communication task. Participants in our study produced sketches depicting the same object (left) over a series of eight blocks (right). Images of objects were rendered from 3D mesh models in the ShapeNet database and appear in this figure with permission.

To test these predictions, we developed a drawing-based reference game where two participants repeatedly produced drawings to communicate the identity of objects in context (see Fig. 1). Our experimental approach builds on pioneering work investigating the emergence of graphical symbol systems and the importance of social feedback for establishing conventional meaning^21,22,36–42^, which belong to a broader literature studying ad hoc convention formation in spoken language^43,44^, written language^45^ and gesture^46–48^. However, our experiments differ in three important ways from that work. First, our work is motivated by different theoretical questions. In particular, our primary goal is to understand the cognitive constraints that enable individual sketchers and viewers to determine the correspondence between pictures and their visual referents in context, rather than the question of where symbols come from or how symbols evolve as a consequence of cultural transmission. Towards this end, we employed a task paradigm in which people used pictures to communicate about visual objects, unlike much prior work that has employed text or music as the target of communication^21,37^.

Second, our work makes several methodological contributions that advance our understanding of pictorial meaning. While related ideas concerning the role of iconicity and convention have been explored in earlier work in experimental semiotics^21,39,42^, methodological limitations in this work temper the conclusions that can be drawn from existing evidence. Our approach addresses these limitations in three key ways. In order to distinguish between task-general and item-specific changes in how people depict objects over time, we use a pre-post design and include a control set of objects. In addition, in order to measure how strongly resemblance drives recognition in the absence of shared interaction history, we recruit naive participants to guess the intended referent of each drawing. Moreover, we leverage recently developed model-based techniques^9,49^ and crowdsourcing to systematically analyze the perceptual similarity between drawings, as well as between drawings and their referents. Both of these techniques provide well validated ways of measuring key aspects of visual resemblance that go beyond the low-level measures of visual complexity used in prior work.

Third, the insights gained from our approach support a view of pictorial meaning that is distinct from that advanced by prior work using similar iterated communication paradigms. Earlier studies suggested that graphical communication may initially rely on iconic relationships between a picture and its referent, but then “drift towards the arbitrary”^40^. By contrast, the evidence we present suggests instead that resemblance continues to exert an influence on how drawings evolve throughout an interaction, with visual elements that are most useful for distinguishing a referent from other objects in context being more likely to be preserved.

## Results

**Fig. 2:**
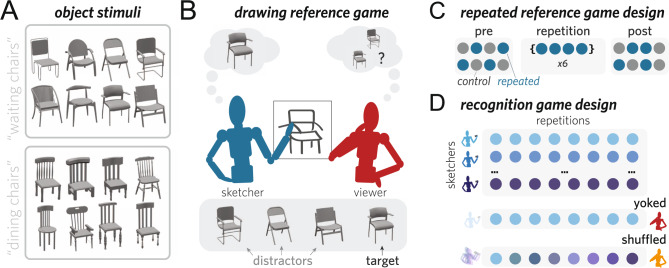
Experimental design. **A** Two object collections were used, each containing eight similar objects. Images of objects were rendered from 3D mesh models in the ShapeNet database and appear in this figure with permission. **B** Pairs of participants performed a drawing-based reference game in which one participant (sketcher) was cued to draw the target object such that the other participant (viewer) could identify it in context. **C** Four objects were drawn repeatedly throughout the interaction; the remaining four control objects were drawn once each at the beginning and end of each interaction. **D** Recognition participants aimed to identify the target object in context based on drawings from the reference game experiment. These drawings were either all from a single communicative interaction (yoked) or from all different interactions (shuffled).

To investigate the potential role that both visual information and shared associations play in determining how people communicate about visual objects, we used a drawing-based reference game paradigm. On each trial, both participants shared a visual context, represented by an array of four objects that were sampled from a set of eight visually similar objects (Fig. 2A). One of these objects was privately designated as the target for the sketcher. The sketcher’s goal was to draw the target so that the viewer could select it from the array of distractor objects as quickly and accurately as possible. Importantly, sketchers drew the same objects multiple times over the course of the experiment, receiving feedback about the viewer’s response after each trial (Fig. 2B). This repeated reference-game design thus allowed us to track both changes in how well each dyad communicated, as well as changes in the content of their drawings over time.

### Improvement in communicative efficiency across repetitions

Given that the focus of our study was on changes in communication behavior over time, we first sought to verify that dyads were able to successfully communicate. We found that even the first time sketchers drew an object, viewers correctly identified it at rates well above chance (proportion correct: 76%, chance = 25%), suggesting that they were engaged with the task but not yet at ceiling performance. In order to measure how well dyads learned to communicate throughout the rest of their interaction, we used a measure of communicative efficiency the *balanced integration score* or BIS ^50^; that takes both accuracy (i.e., proportion of correct viewer responses) and response time (i.e., latency from beginning of trial until viewer response) into account. This efficiency score is computed by first *z*-scoring accuracy and response time for each drawing within an interaction, in order to map different interactions onto the same scale. We then combined these measures by subtracting the standardized response time from standardized accuracy. Efficiency is highest when dyads are both fast and accurate, and lowest when they make more errors and take longer, relative to their own performance on other trials.

**Fig. 3:**
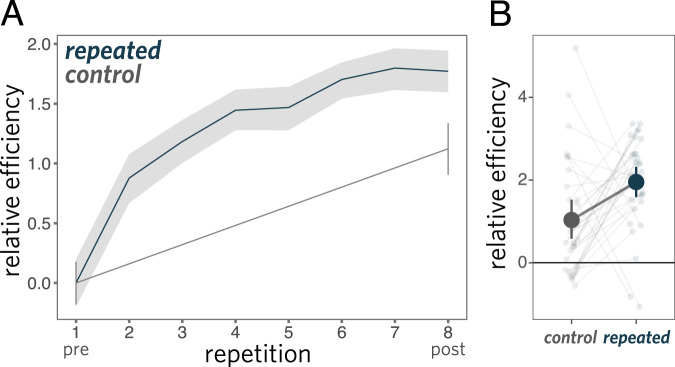
Performance on interaction communication experiment. Communication efficiency **A** across repetitions and **B** from the pre to post phase, where each line shows the effect for a given dyad. Efficiency combines both speed and accuracy, and is plotted relative to the first repetition. Error ribbons and bars represent 95% CI.

**Fig. 4:**
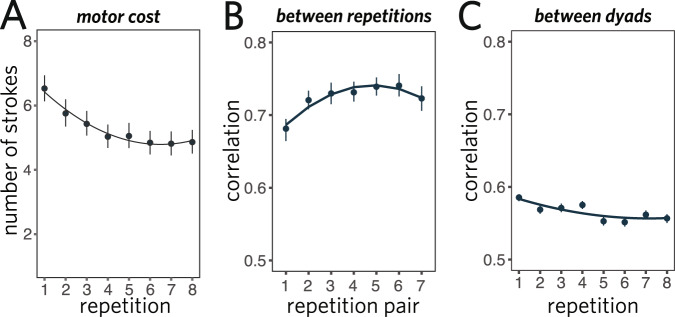
Feature analysis for sketches produced during communication task. **A** Decrease in number of strokes used to produce drawings across repetitions. **B** Increased consistency between successive drawings throughout an interaction. **C** Increased dissimilarity between drawings of same object from different interactions. Error bars represent 95% CI.

We fit a linear mixed-effects model predicting efficiency as a function of repetition block, including random slopes for each dyad. We treat repetition block as a continuous predictor (integers 1 through 8); to account for non-linearities in the effect, we also included an orthogonalized quadratic effect of repetition (see Supplementary Methods for a Bayesian mixed-effects model making the weaker assumption of monotonicity). We found that communicative efficiency reliably improved across repetitions of each object (*b* = 23.5, *t*(66) = 13.5, *p* < 0.001; Fig. 3). Similar improvements were found when examining only raw response times (12.2s on first repetition, 6.8s on final repetition: *b* = − 69.9, *t*(66) = − 11.5, *p* < 0.001) or raw accuracy (76% on first repetition, 90% on final repetition: *b* = 26.6, *z* = 5.8, *p* < 0.001 in logistic regression), indicating that participants had achieved greater efficiency by becoming both faster and more accurate. One straightforward explanation for these gains is that sketchers were able to use fewer strokes per drawing to achieve the same level of viewer recognition accuracy. Indeed, we found that the number of strokes in drawings of repeated objects decreased steadily as a function of repetition (*b* = −22.9, *t*(66) = −6.00, *p* < 0.001; Fig. 4A). Overall, these results show that dyads were able to visually communicate about these objects more effectively across repetitions.

### Object-specific improvements in communicative efficiency

While these performance gains are consistent with the possibility that participants had developed ways of depicting each object that were dependent on previous attempts to communicate about that object, these gains may also be explained by general benefits of task practice. To tease apart these potential explanations, we also examined changes in communication performance for a set of control objects that were drawn only once at the beginning (pre phase) and at the end (post phase; Fig. 2C). In the pre phase, there was no difference in accuracy between repeated and control objects (75.7% repeated, 76.1% control), which was expected, as objects were randomly assigned to repeated and control conditions. To evaluate differential changes in communicative efficiency across conditions, we fit a linear mixed-effects model including categorical fixed effects of phase (pre vs. post), condition (repeated vs. control), and their interaction, as well as random intercepts, slopes, and interaction coefficients for each dyad. We found that communicative efficiency reliably increased overall between the pre and post phases (*b* = 0.72, *t*(137) = 14.6, *p* < 0.001), suggesting at least some general benefit of task practice. Critically, however, we also found a reliable interaction between phase and condition: communicative efficiency improved to a greater extent for repeated objects than control objects (*b* = −0.16, *t* = − 3.17, *p* = 0.002; see Fig. 3). To account for possible artifacts introduced by imbalances in the number of repeated and control trials in the *z*-scoring procedure, we also ran the same analysis *z*-scoring only within the balanced set of 16 pre and post trials. The effect was similar (*b* = −0.14, *t* = −3.10, *p* = 0.002). We also note that post-test performance in the control condition is comparable to that in the 2nd repetition block of the repeated condition (82.8% and 83.2% accuracy, respectively), although response times were somewhat faster on the post-test, consistent with general task-practice effects. These improvements in efficiency are due to both changes in raw (un-transformed) accuracy (control: +7.1%, repeated: +14.5%; interaction: *b* = − 0.46, *z* = −2.8, *p* = 0.005 using logistic regression) and raw response time (control:−3.8s, repeated: −5.3s; interaction: *b* = 0.38, *t* = 2.8, *p* = 0.006). See Supplementary Methods A2.2 for further analyses. Together, these data provide evidence for benefits of repeatedly communicating about an object that accrue specifically to that object.

An intriguing possibility is that dyads achieved such benefits by developing ad hoc graphical conventions establishing what was sufficient and relevant to include in a drawing to support rapid identification of objects they repeatedly communicated about. To investigate this possibility, we examined how the drawings themselves changed throughout each interaction, hypothesizing that successive drawings of the same object produced within an interaction changed less over time as dyads converged on consistent ways of communicating about each object. For these analyses, we capitalized on recent work validating the use of image features extracted by deep convolutional neural network (DCNN) models to measure visual similarity between drawings^9^. Specifically, we used a DCNN architecture known as VGG-19^49^ to extract feature vectors from pairs of successive drawings of the same object made within the same interaction (i.e., repetition *k* to *k* + 1), and computed the correlation between each pair of feature vectors. A mixed-effects model with random intercepts and slopes for both target object and sketcher revealed that the similarity between successive drawings increased throughout each interaction (*b* = 0.62, *t*(12) = 3.84, *p* = 0.002; Fig. 4B), providing support for the notion that dyads converged on increasingly consistent ways to communicate about each object (see Supplementary Note 1 for additional control analyses demonstrating that these effects are not driven by lower-level features such as the amount of empty space on the drawing canvas).

### Effect of shared interaction history on performance

One way of understanding our results so far is that the need to repeatedly refer to certain objects is sufficient to explain how the way sketchers depicted them changed over time. However, these objects did not appear in isolation, but rather as part of a communicative context including a consistent viewer and the other, distractor objects. How did this communicative context influence the way drawings conveyed meaning about the target object across repetitions? To investigate this question, we conducted a follow-up recognition experiment (see Fig. 2D) including two control conditions to estimate how recognizable these drawings were to naive viewers, outside the communicative context in which they were produced. Participants in the *shuffled* control group were shown a sequence of drawings spliced together from many different interactions, thus disrupting the continuity experienced by viewers paired with a single sketcher. Because this offline recognition task differed in several ways from the interactive communication task, we also included a more directly comparable *yoked* control group who were shown a sequence of drawings taken from a single interaction, closely matching the experience of viewers in the communication experiment One noteable difference is that viewers in our main communication experiment saw the drawing unfold stroke-by-stroke in real-time whereas viewers in the recognition experiment were only shown the finished drawing as a static image. We address this difference in an internal replication discussed below (see Supplementary Methods, A2.3).

Insofar as interaction-specific shared associations contributed to the efficiency gains observed previously, we hypothesized that the *shuffled* group would not improve as much over the course of the experimental session as the *yoked* (or original *communication*) group would. Both conditions preserved the repetition index of the original drawing, so a drawing that was produced in the 5th repetition block of a communication game was presented in the 5th repetition block of the shuffled and yoked conditions; this manipulation is importantly different from prior studies which manipulated the order in which trials were played back to naive comprehenders e.g., ^51^, who reversed the trial order or omitted early trials altogether e.g ^52,53^, who dropped naive comprehenders into later blocks. Critically, groups in both control conditions received exactly the same amount of practice recognizing drawings, and performed the task under the same incentives to respond quickly and accurately; the only difference between conditions was whether the drawings had been produced by the same sketcher. Thus any differences in performance between these groups is attributable to the impact of interaction history on a drawing’s meaning.

**Fig. 5:**
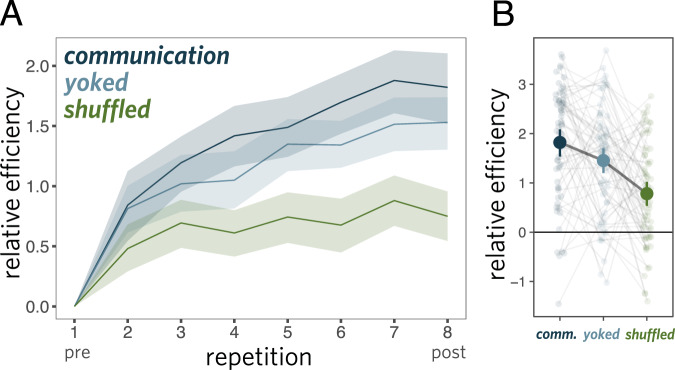
Performance on recognition experiment. **A** Comparing drawing recognition performance between viewers in communication experiment (dark blue) with those of yoked (light blue) and shuffled (green) control groups. **B** Individual variation in performance on the final repetition in the post phase (relative to that in the pre phase), where grey lines connect drawings originally produced by the same sketcher. Error ribbons and bars represent 95% CI.

We compared the yoked and shuffled groups by measuring changes in recognition performance across successive repetitions using the same efficiency metric we previously used. We estimated the magnitude of these changes by fitting a linear mixed-effects model that included group (yoked vs. shuffled), repetition number (i.e., first through eighth), and their interaction, as well as random intercepts and slopes for each participant. While we found a significant increase in recognition performance across both groups (*b* = 0.18, *t* = 12.8, *p* < 0.001), we also found a large and reliable interaction: yoked participants improved their efficiency to a substantially greater degree in than shuffled participants (*b* = 0.10, *t* = 4.9, *p* < 0.001; see Fig. 5). Analyzing accuracy (yoked: +15.8%, shuffled: +5.6%; interaction: *b* = 0.62, *z* = 2.8, *p* = 0.0046 in logistic regression) or response time (yoked: -1.6s, shuffled: -1.1s, interaction: *b* = −0.14, *t* = −4.8, *p* < 0.001) alone yielded similar results: the yoked group improved to a greater degree across each experimental session and practice effects observed on the set of control objects were comparable (see Supplementary Fig. S2A).

Taken together, these results suggest that third-party observers in the yoked condition who viewed drawings from a single interaction were able to take advantage of this continuity to more accurately identify what successive drawings represented. While observers in the shuffled condition still improved somewhat over time, being deprived of this continuity made it relatively more difficult to interpret later drawings. These findings are consistent with prior work while additionally controlling for confounds present in earlier studies, including task practice^21^, which we were able to equate between the shuffled and yoked conditions. (While not central to the questions posed in the current studies, it would be possible to further disentangle the impact of task practice from the effect of repetition on recognition performance by including another control condition in which observers are presented with a randomized sequence of drawings from the same interaction.)

Such results could arise if early drawings were more strongly constrained by the visual properties of a shared target object, but later drawings diverged as different dyads discovered different equilibria in the space of viable graphical conventions. Under this account, drawings of the same object from different dyads would become increasingly dissimilar from each other across repetitions. We again tested this prediction using high-level visual representations of each drawing derived from a deep neural network. Specifically, we computed the mean pairwise similarity between drawings of the same object within each repetition index, but produced in different interactions. In other words, we considered all interactions in which a particular object was repeatedly drawn, then computed the average similarity between drawings of that object made by different sketchers at each point in the interaction. In a mixed-effects regression model including linear and quadratic terms, as well as random slopes and intercepts for object and dyad, we found a small but reliable negative effect of repetition on between-interaction drawing similarity (*b* = −1.4, *t* = −2.5; Fig. 4C). We also conducted a permutation test to compare this *t* value with what would be expected from scrambling drawings across repetitions for each sketcher and target object and found that the observed slope was highly unlikely under this distribution (95% *CI* = [−0.57, 0.60], *p* < 0.001). Taken together, these results suggest that drawings of even the same object can diverge over time when produced in different communicative contexts.

Unlike viewers in the interactive visual communication experiment, participants in the yoked condition made their decision based only on the whole drawing and were unable to interrupt or await additional information if they were still uncertain. As such, we conducted an exploratory analysis comparing the yoked group against the original communication group to estimate the contribution of these (minimal) viewer feedback channels to gains in performance^21,53^. Because the feedback channel in our task was lower in bandwidth than typically used in prior studies, we expected the gap between interactive viewers and offline viewers to be relatively small. In a mixed-effects model with random intercepts, slopes, and interactions for each unique trial sequence, we found a strong main effect of repetition (*b* = 0.23, *t* = 12.8, *p* < 0.001), as well as a weaker but reliable interaction with group membership (*b* = −0.05, *t* = −2.2, *p* = 0.032; Fig. 5), showing that the yoked group improved at a slightly more modest rate than viewers in the original communication experiment had. To better understand this interaction, we further examined changes in the accuracy and response time components of the efficiency score (see Supplementary Fig. S2A). We found that while viewers in the communication experiment were more accurate than yoked participants overall (communication: 88%, yoked: 75%; main effect: *b* = 0.90, *z* = −6.0, *p* < 0.001 in logistic regression), improvements in accuracy were similar in both groups (communication: +15.9%, yoked: +15.8%). The interaction instead appeared to be driven by reductions in response time between the first and final repetitions (communication: 10.9s to 5.84s; yoked: 4.66s to 3.31s). Unsurprisingly, response times were shorter overall in the yoked group, given that these participants did not need to wait for the drawing to be produced before making a decision. Their response times may thus already have been closer to floor, limiting the possible reduction in response time. (We found no differential change in performance across yoked and communication groups in our internal replication, after removing the ability for the viewer to interrupt the sketcher. On the one hand, closing the gap between the yoked and communication conditions made the primary yoked vs. shuffled comparison more meaningful in the replication; on the other hand, because feedback was restricted purely to the viewer’s guess, the sketcher’s drawings may be less viewer-dependent in this variant).

### Sketchers preserve visual properties that are diagnostic of object identity

**Fig. 6:**
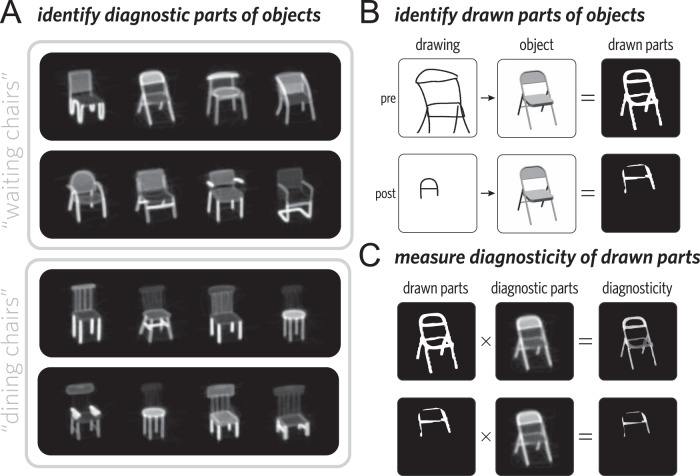
Analysis pipeline for evaluating visual diagnosticity. **A** Annotators indicated which parts of an object were most diagnostic in context (brighter regions are more diagnostic), yielding a graded diagnosticity heatmap for each object. **B** A separate group of annotators also indicated which parts of objects were depicted in each drawing, yielding a binary image mask for each drawing. Images of objects were rendered from 3D mesh models in the ShapeNet database and appear in this figure with permission. **C** Mean diagnosticity for a drawing was computed by averaging the diagnosticity values of all pixels in the object diagnosticity map that appeared in that drawing.

Our results in the previous section suggest that viewers depend on a combination of visual information and socially mediated associations to successfully recognize drawings. Specifically, we found that it was increasingly difficult for viewers in the shuffled condition to make sense of drawings in the absence of shared interaction history with the same communication partner. While these findings focused primarily on the cognitive mechanisms employed by the viewer, the increasing sparsity of the drawings suggest that decisions about drawing production may also be guided by a combination of visual information and social context. In this section we ask: Why was some visual information preserved during the formation of these graphical conventions while other information was not? One possibility is that these choices are mostly arbitrary: given a sufficiently long interaction history to establish the association, any scribble could in principle be used to refer to any object. An alternative possibility is that these choices are systematically driven by the visual information conveyed by each pen stroke: sketchers may preserve information about diagnostic or salient parts of the target object, rather than omitting visual information in an arbitrary fashion. For example, in the contexts shown in Fig. 6A, the folding chair (top row, second from left) has a seat that is similar to the distractors, but a distinctive backrest and set of legs. If sketchers are under pressure to produce informative drawings for their partner in context^20,45^, their later drawings may come to reflect these pressures.

To test this hypothesis, and obtain reliable estimates of what information was diagnostic in context, we required a large number of drawings for a smaller set of contexts. Instead of randomly sampling different contexts for each dyad, as before, we adapted our reference-game paradigm to only include two pre-generated contexts for every dyad, which were counterbalanced across the repeated and control conditions. We also made one important modification to our experimental design to address a potential confound. Rather than allowing the viewer to interrupt the sketcher with an early response, we required the sketcher to click a button once they were ready to show their drawing to the viewer. Here, drawing duration is purely a function of the sketcher’s independent decisions about what needs to be included in a drawing, whereas in our original design, it was a joint combination of the sketcher’s decision and the viewer’s decision threshold for when to interrupt. That is, it was possible in the original design that any apparent effects of conventionalization were purely driven by the viewer, with the sketcher simply following a heuristic to continue adding more detail until the viewer made a decision. This modification prevents such a strategy. Aside from these changes, the design was identical to the original repeated reference game.

We recruited a sample of 65 additional dyads (130 participants) for this task. In addition to providing sufficient power for our diagnosticity analyses, this new sample also provided an opportunity to conduct an internal replication to evaluate the robustness of our results (see Supplementary Methods for successful replications of our earlier analyses on these new data). Next, we recruited a separate sample of naive annotators to provide judgments concerning the diagnosticity of each element in these drawings. Using annotations in this way is based on the assumption that people are capable of accurately judging both how individual elements of drawings correspond to parts of an object and what information would be diagnostic of object identity when performing the viewer task. One group of annotators indicated which parts of objects were depicted in each drawing by painting over the corresponding regions of the target object image (Fig. 6B), yielding a binary image mask for each drawing. A second group of annotators indicated which parts of objects were most diagnostic in context by painting over regions of each target object that distinguished it the most from each distractor object, yielding a graded heat map of diagnostic regions over each object (Supplementary Fig. S3).

**Fig. 7:**
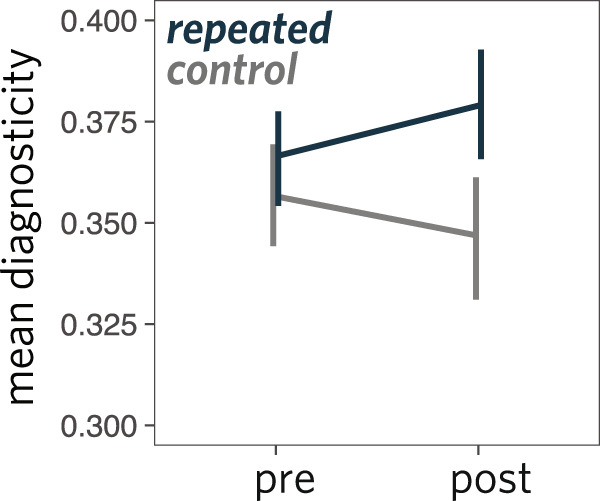
Results for diagnosticity analysis. Changes in the mean diagnosticity of drawn parts is shown from the pre to post. Error bars represent bootstrapped 95% confidence intervals.

To measure changes in the diagnosticity of drawings over time, we took the intersection of these annotation maps for each drawing (see Fig. 6C). We then took the average diagnosticity value per pixel in the combined stroke map to control for the overall size of the drawing, yielding a metric reflecting how much the sketcher had selectively prioritized diagnostic parts of the object overall. Our primary hypothesis concerned differential changes in diagnosticity over time. Insofar as new graphical conventions are shaped by communicative context, gradually depicting the most distinctive regions of the image while omitting less distinctive regions, we predicted that the repeated drawings would increase in diagnosticity between the pre and post phases. Meanwhile, to the extent that these changes in diagnosticity depend on having communicated repeatedly about an object, we predicted that the diagnosticity of control drawings would remain stable over time. To test these hypotheses, we conducted a mixed-effects regression analysis on diagnosticity values for each drawing. We included fixed effects of phase (pre vs. post) and condition (repeated vs. control) as well as their interaction. While the maximal random effects structure did not converge, we were able to include intercepts and main effects for each sketcher and each target object. Consistent with our hypothesis, we found a significant interaction (*b* = −0.05, *t* = −3.4, *p* < 0.001, Fig. 7): objects in the repeated condition became increasingly diagnostic as they became sparser, relative to those in the control condition. More broadly, together with the earlier model-based analyses, these findings provide converging evidence that drawings were changing in contentful ways throughout an interaction, reflecting both systematic biases towards object-diagnostic information, as well as variability across dyads with respect to which aspects of this information to preserve.

## Discussion

The puzzle of pictorial meaning has long resisted reductive explanations. Classical theories have either argued that a picture’s meaning is primarily determined by visually resembling entities in the world, or by appealing to socially mediated conventions. However, these theories fail to explain the full range of pictures that people produce. In this paper, we proposed an integrative cognitive theory where both resemblance and conventional information jointly guide inferences about what pictures mean. We evaluated this theory using a Pictionary-style communication game in which pairs of participants developed novel graphical conventions to depict objects more efficiently over time. Our theory predicted that viewers would initially rely on visual resemblance between the drawing and object to successfully determine the intended referent, but rely increasingly on experience from earlier communicative exchanges even as direct resemblance decreased. We tested these predictions by manipulating the amount and type of socially mediated experience available to the viewer: we varied how often each object had been drawn throughout an interaction and whether the drawings were produced by the same individual. We found that viewers improved to a greater degree for objects that had been drawn more frequently; conversely, viewers had greater difficulty recognizing sequences of drawings produced by different individuals. We further tested the prediction that sketchers in our task would also increasingly rely on shared experience with a specific viewer, and found that people produced progressively simpler drawings that prioritized the most diagnostic visual information about the target object’s identity. Taken together, our findings suggest that visual resemblance forms a foundation for pictorial meaning, but that shared experiences promote the emergence of depictions whose meanings are increasingly determined by interaction history rather their visual properties alone.

**Fig. 8:**
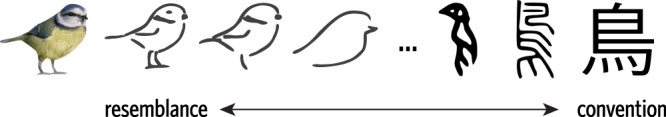
Schematic of pictorial meaning as a spectrum. Our findings support the notion that both visual resemblance and socially mediated conventions jointly guide inferences about pictorial meaning. Leftmost color image was rendered from 3D mesh model licensed by TurboSquid.

There are several important limitations of the current work that future studies should address to further evaluate this integrative theory of pictorial meaning (see Fig. 8). First, here we focused on how people use drawings to communicate about the identity of a visual object from a single real-world object category (i.e., chairs). Thus it is not yet clear to what degree the current findings generalize to visual communication behavior in other settings. While there is reason to think these findings would generalize to other categories, given recent findings employing a similar methodology using multiple categories to reveal the influence of social context^20^, a natural opportunity for future work is to directly evaluate the same questions for a broader array of objects. There are other aspects of our methodological approach that offered concrete benefits, but could be fruitfully adapted to serve open questions in future work. For example, by using photorealistic 3D object renderings as stimuli, we were able to leverage recently developed model-based techniques for encoding high-level visual features of both drawings and objects into the same latent feature space to measure key aspects of visual resemblance^9^. Moreover, focusing on exemplars appearing in a consistent pose from a single object category helped to isolate key sources of drawing variability of primary interest, namely: the visual properties that were most diagnostic of an object’s identity and the accumulation of shared context between communicators over time. Nevertheless, people also produce pictures to communicate about non-visual concepts, such as semantic associations^21,54^, narrative information^55,56^, number^57,58^, and causal mechanisms^59–62^.

It is currently unknown whether the same general-purpose visual processing mechanisms will be sufficient to explain how graphical conventions emerge to convey these more abstract concepts. To the extent that general-purpose visual encoding models can easily generalize to a particular ‘non-visual’ concept without relying upon ad hoc associative learning, then visual resemblance may play a stronger role in explaining how that abstract concept is grounded in graphical representations of them. On the other hand, if and when such associative learning mechanisms are necessary above and beyond such generic visual processing mechanisms to explain the mapping between a picture and an abstract concept (e.g., “42” or →), then conventionality may play a stronger role for explaining how such pictures become meaningful in context, consistent with existing descriptive accounts of what distinguishes symbols from icons^63–66^. Substantial mechanistic clarity may be gained by developing more robust computational models that can operate on a broader range of images to predict a greater variety of abstract meanings beyond the identity of individual objects. Moreover, people produce pictures for other purposes than to communicate efficiently about concepts — indeed, drawings can also induce an aesthetic response^67^ and be used for emotional regulation^68,69^. An important direction for future work would thus also be to investigate the relationship between the cognitive processes involved in drawing-based communication and the full set of psychological mechanisms involved during the production and interpretation of drawings in these other contexts.

Another opportunity for future work comes from observing that providing the viewer with the ability to interrupt the sketcher was associated with greater communicative efficiency. While yoked viewers in our main communication experiment achieved lower accuracy than did viewers participating in the reference game, viewers in our internal replication study who could not interrupt sketchers did not outperform their corresponding yoked viewers. These findings are consistent with the notion that even simple forms of social feedback during real-time communication can be impactful, consistent with prior work^21^. These results also raise important questions concerning the relationship between this feedback and the information conveyed in subsequent messages in an extended communicative interaction. For example, supposing that the viewer used the timing of their guess to signal that the final stroke produced by the sketcher was the most diagnostic, the sketcher might be able to leverage that cue to prioritize that information in subsequent drawings. More generally, supposing that the last stroke provides information that incrementally serves to increase the viewer’s belief that some object was the target, future studies could employ drift-diffusion processes to model the time course of viewer decision making. Such models would posit that viewers gradually accumulate evidence before reaching some decision threshold and making their selection, and a sketcher model that reasons about this diffusion process could explain the inferences that are supported by viewer feedback^70^.

Another important direction for future work is to explore why drawings are produced at different points along the resemblance-convention continuum at all. In other words, if resemblance is sufficient, why rely upon socially mediated experience at all? Our paradigm suggests that production cost may be one important factor driving such behavior. Recent computational models of visual communication have found that how costly a drawing is to produce (i.e., in time/ink) is critical for explaining the way people spontaneously adjust the level of detail to include in their drawings in one-shot visual communication tasks^20^. We expect that the consequences of this intrinsic preference for less costly drawings may be compounded across repetitions effectively increasing the capacity of the communication channel, consistent with findings from non-visual communication modalities^71^. In other words, the accumulation of feedback and interaction history allows people to continue to be informative with fewer strokes. The magnitude of such implicit production costs may vary across individuals, however, motivating our use of explicit incentives for all participants to complete trials efficiently. For example, different dyads may adopt different drawing “styles” that are orthogonal to informational content, but demand more or less effort or skill. Further work on pictorial meaning should explore other considerations driving the tradeoffs between relying on resemblance-based and convention-based cues, including the reliability of resemblance-based information, the complexity of the target concept, and the availability of social feedback e.g., ^72,73^.

Finally, the framework we have proposed for understanding pictorial meaning may help illuminate why visual communication has been such a uniquely powerful vehicle for the cultural transmission of knowledge across so many cultures. In particular, our work suggests that the ability to easily rely on resemblance-based cues to meaning gives the visual modality distinct advantages over other modalities for conveying certain information. In other words, the cognitive mechanisms supporting successful visual communication may be rooted both in general-purpose visual processing mechanisms that are broadly shared by humans, facilitating communication between members of different language communities even in the absence of shared graphical conventions. At the same time, our work also generalizes several insights from theories originally developed to account for communicative convention formation in language, but whose inference mechanisms have been proposed to apply generally across communication modalities^20,74^. Advancing our knowledge of the cognitive mechanisms underlying pictorial meaning may thus lead to a deeper understanding of how humans are capable of seamlessly integrating such a huge variety of graphical and symbolic representations to think and communicate.

## Methods

### Communication experiment

#### Participants

For our main communication experiment, we recruited 138 participants from Amazon Mechanical Turk, who were paired up to form 69 dyads to play a drawing-based reference game^75^. To provide sufficient power for our diagnosticity analyses, we needed to collect a larger number of observations from a smaller number of specific contexts, so we recruited an additional 130 participants (66 dyads) to participate in a follow-up experiment (which also served as an internal replication). Participants in both experiments were provided a base compensation of $1.50 for participation and were able to earn an additional $1.60 in bonus pay based on task performance. On average, participants received $1.13 in bonus payments for completing this 20-minute experiment, thereby earning approximately $7-$8 per hour. In this and subsequent experiments, participants provided informed consent in accordance with the Stanford IRB.

#### Stimuli

In order to make our task sufficiently challenging, we sought to construct visual contexts consisting of objects whose members were both geometrically complex and visually similar. To accomplish this, we sampled objects from ShapeNet^76^, a public database containing a large number of 3D mesh models of real-world objects. We restricted our search to 3096 objects belonging to the chair class, which is among the most diverse and abundant in ShapeNet. To identify groups of visually similar objects, we employ neural-network based encoding models to extract high-level feature representations of images. Specifically, we used the PyTorch implementation of the VGG-19 architecture pre-trained to perform image classification on the ImageNet database^49,77,78^, an approach that has been validated in prior work to provide a reasonable proxy for human perceptual similarity ratings between images of objects^79,80^. This feature extraction procedure yields a 4096-dimensional feature vector for each rendering, reflecting activations in the second fully-connected layer (i.e., fc6) of VGG-19, a higher layer in the network. We then applied dimensionality reduction (PCA) and *k*-means clustering on these feature vectors, yielding 70 clusters containing between 2 and 80 objects each. Among clusters that contained at least eight objects, we manually defined two object categories containing eight exemplars each, which roughly correspond to ‘dining chairs’ and ‘waiting-room chairs.’

#### Experimental design

For each dyad in the main communication experiment, two sets of four objects were sampled from the object categories derived above, defining two communication contexts: one was designated the *repeated* set while the other served as the *control* set. (In half of the dyads, the four control objects were from the same visual category as repeated objects; in the other half, they were from different categories within the same basic-level category (i.e., chairs). Only objects from either the ‘dining’ and ‘waiting-room’ categories were used as stimuli in the current studies. The rationale for using both sampling strategies was to support investigation of between-cluster generalization in future analyses. In our current analyses, we collapse across these groups.) In our follow-up communication experiment (a component of our internal replication), instead of randomly sampling objects from all ‘waiting-room’ and ‘dining’ chairs, we defined two fixed sets of four objects for all participants — one set of four ‘waiting-room’ chairs and one set of four ‘dining’ chairs. The assignment of these sets to the *repeated* and *control* conditions were counterbalanced across participants.

The experiment consisted of three phases: a pre, repetition, and post phase. During the *repetition* phase, there were 24 trials: six repetition blocks of four trials, with each of the four repeated objects appearing as the target once in each repetition block. In each of the pre and post phases, there were eight trials, with each repeated and control object appearing once as targets (in their respective contexts) in a randomly interleaved order. The full experimental session thus consisted of a sequence of 40 trials (i.e., 8 pre + 24 repetition + 8 post), with *repeated* objects serving as the target a total of eight times, and *control* objects serving as the target twice (i.e., once at the beginning and once at the end).

#### Task procedure

In each dyad, one participant was assigned the sketcher role and the other was assigned the viewer role. These role assignments remained the same throughout the experiment. On each trial, both participants were shown the same set of four objects in randomized locations. One of the four objects was highlighted on the sketcher’s screen to designate it as the target. Sketchers drew using their mouse cursor in black ink on a digital canvas embedded in their web browser (300 × 300 pixels; pen width = 5px). Each stroke was rendered on the viewer’s screen in real time and sketchers could not delete previous strokes. The viewer aimed to select the true target object from the context of four objects as soon as they were confident of its identity, and both participants received immediate feedback: the sketcher learned when and which object the viewer had clicked, and the viewer learned the true identity of the target. Participants were incentivized to perform both quickly and accurately. They both earned an accuracy bonus for each correct response, and the sketcher was required to complete their drawings in 30 seconds or less. If the viewer responded correctly within this time limit, participants received a speed bonus inversely proportional to the time taken until the response.

Only one procedural difference was introduced for our internal replication. Instead of allowing the viewer to interrupt the production of the drawing at any point (as in Pictionary), we asked the viewer to wait until the sketcher decided to finish drawing and press a “Done” button. We then showed the completed drawing to the viewer as a static image instead of animating in each stroke individually. This change removed potential confounds between the sketcher’s decision-making and the viewer’s decision-making, as the drawing time was now purely under the sketcher’s control. Additionally, presenting static drawings also provided a closer match between the experience of viewers in the communication experiment and that of participants in the recognition experiments.

#### Exclusion criteria

We took several measures to ensure that participants were attentive and understood the task. First, participants were only paired up with a partner once they passed a comprehension quiz covering important points from the instructions. Second, upon completion of data collection, we flagged and excluded games where participants either repeatedly timed out (i.e., where the sketcher repeatedly failed to make a drawing or the viewer repeatedly failed to make a selection), did not appropriately follow instructions (e.g., using the drawing canvas to write out text, or producing inappropriate drawings), or were a significant outlier on key metrics (e.g., having used an unusually large number of strokes or having achieved unusually low accuracy). Using these criteria, we excluded one game from our main communication experiment and four games from the follow-up experiment (i.e., internal replication). Nevertheless, the decision to include/exclude data from these sessions did not substantively impact our key findings. Third, if one participant disconnected for any reason prior to completion of the task (e.g., by closing their browser tab or losing their internet access), the other participant was immediately directed to the exit survey and awarded the bonus they had earned up to that point. Of the 73 games that were started in our original task, only five disconnected; in our replication, only six of the 76 games disconnected.

### Recognition experiments

#### Participants

In our main set of recognition experiments, we recruited 245 participants via Amazon Mechanical Turk and excluded data from 22 participants who did not meet our inclusion criteria for accurate and consistent response on attention-check trials, leaving a sample of 223 participants (i.e., 106 in *yoked*, 117 in *shuffled*). We also conducted follow-up recognition experiments accompanying the second set of reference-game data collected to enable our diagnosticity analyses and provide an internal replication. In these experiments, we obtained data from an additional 225 participants, after exclusions (i.e., 100 in *yoked*, 125 in *shuffled*).

#### Experimental design & task procedure

On each trial, participants were presented with a static rendering of a completed drawing and the same set of four objects that accompanied that drawing in the original visual communication experiment. They were then asked to indicate which of the four objects they believed the drawing was intended to represent. They also received exactly the same feedback, as well as the same accuracy and speed-related incentives as in the communication experiment. To ensure task engagement, we included five identical attention-check trials that appeared once every eight trials. Each attention-check trial presented the same set of objects and drawing, which we identified during piloting as the most consistently and accurately recognized by naive participants. Only data from participants who responded correctly on at least four out of five of these trials were retained in subsequent analyses. Each participant was randomly assigned to one of two conditions: a *shuffled* group and a *yoked* group.

Participants in the *shuffled* group were shown a sequence of drawings spliced together from many different interactions, thus disrupting the continuity experienced by viewers paired with a single sketcher. Specifically, each *shuffled* participant viewed drawings from 10 different interactions in the original communication experimental cohort. Four drawings came from each of those interactions, one drawing of each object in a visual context. Each set of four drawings appeared in the same repetition block as it had originally, such that drawings appeared at approximately the same time point in the recognition experiment as they had in the original communication experiment. For example, if a drawing was produced in the 5th repetition block in the original experiment, then it also appeared in the 5th repetition block for *shuffled* participants.

Because the recognition task differed in several ways from the interactive communication task, we also included a more directly comparable *yoked* control group who were shown a sequence of drawings taken from a single interaction, closely matching the experience of viewers in the communication experiment while also being directly comparable to the experience of the *shuffled* group. Participants in the *yoked* group viewed all 40 drawings from a single interaction in the communication experiment in the same sequence that the original viewer had. Thus the key difference between the *yoked* and *shuffled* groups was whether drawings came from the same sketcher, preserving continuity in communicative context across repetition blocks, or whether drawings came from a large number of different sketchers, disrupting that continuity.

### Model-based analyses of drawing features

To extract high-level visual features of drawings, we used the same PyTorch implementation of the VGG-19 architecture that we used to cluster our stimuli^49,78^. This feature extraction procedure yields a 4096-dimensional vector representation extracted from the penultimate layer of the neural network (i.e., fc6) for each drawing of every object, in every repetition, from every interaction. Using this feature basis, we compute the similarity between any two drawings as the Pearson correlation between their feature vectors (i.e., ${s}_{ij}=cov({\overrightarrow{r}}_{i},{\overrightarrow{r}}_{j})/\sqrt{var({\overrightarrow{r}}_{i})\cdot var({\overrightarrow{r}}_{j})}$). Using these learned feature representations to approximate human judgments about the high-level visual properties of drawings has been validated in prior work^9,20^. In particular, the features we analyze are taken from deeper layers of VGG-19 that have been shown to provide a more explicit representation of semantically relevant visual features (i.e., about object identity) than pixel-based representations or features from earlier layers^20^. This prior work, when taken together with other work comparing how well VGG-19 performs on independently defined neural and behavioral benchmarks related to visual object recognition^81^, justifies our focus on analyzing features from VGG-19. Nevertheless, there is also reason to believe that other deep convolutional neural network architectures (e.g., AlexNet, ResNet) trained in similar ways could be used instead without substantially impacting our findings^79^. Following prior work^9,20^, we focus our analyses on targeted questions concerning the impact of our experimental manipulations on the amount of task-relevant visual information contained in each drawing. However, we note that such feature representations also support exploratory analyses examining correspondences between particular subsets of these features and particular aspects of each image they are most sensitive^82–84^, a promising avenue for future research.

### Empirical measurement of drawing-to-object correspondences

A major challenge that arises when comparing multiple drawings is the drawing-to-object correspondence problem. Different drawings of the same object may be made at different scales, or translated with some spatial offset on the canvas. Additionally, when different drawings depict different partial views of an object, it is not straightforward to determine how exactly strokes in one drawing should map onto strokes in the other. To address these challenges, we designed a drawing-to-object mapping task that allows all sketches in our dataset to be projected into a common space (see Supplementary Fig. S1A). This task was implemented with a simple annotation interface. On one side of the screen, participants were shown a line drawing. On the other side of the screen, they were shown a paint canvas containing the target object the drawing was intended to depict. For each stroke in the line drawing, participants were asked to paint over the corresponding region of the target object. We highlighted one stroke at a time, using a bright green color to visually distinguish it, and participants clicked “Done” when they were finished making their annotation for that stroke. Participants were not allowed to proceed to the next stroke until some paint was placed on the canvas. To provide context, we also showed participants the history of the interaction in which the drawing appeared, so it would be clear, for instance, that an isolated half-circle corresponds to the top of the back rest, given more exhaustive earlier drawings. They continued through all strokes of the given drawing in this way, and then proceeded to the next drawing, annotating a total of 10 different drawings in a session. We recruited 443 participants from Amazon Mechanical Turk to perform the annotation task. We excluded participants who consistently provided low-quality annotations (i.e., participants who made random marks on the canvas to finish the task as quickly as possible) through a combination of manual examination and response latencies. We continued to recruit until all 2600 drawings in our dataset had at least one high-quality drawing-to-object correspondence map. Finally, to reduce noise from annotators who drew outside the bounds of the image (where diagnosticity was low by definition), we applied a simple masking step in post-processing. Specifically, we extracted a segmentation map from the ground truth image of the object to zero out any pixels in the map that corresponded to the background rather than the object.

### Empirical measurement of object-diagnostic features

We recruited 117 participants from Amazon Mechanical Turk to provide diagnosticity maps for each target object, relative to its context. The task interface was similar to the one we used to elicit drawing-to-object correspondences (see Supplementary Fig. S1B). A target object was displayed on the left side of the screen and a foil was displayed on the right side. Participants were instructed to paint over the parts of the target object that were most distinctive and different from the foil. We elicited pairwise comparisons instead of showing the full context to reduce confusion about what was meant by “most different” (i.e., in a large enough context, every part of an object has some difference from at least one distractor). Each participant provided exactly one response for all 16 target objects used in our fixed-context experiment, and we randomly assigned participants to one of 24 possible permutations of distractors, such that different participants saw each target object paired with different distractors. This yielded at least 30 ratings for each pair of objects. To create our final heat maps (as shown in Fig. 6A), we aggregated diagnosticity ratings across the three possible foils in post-processing by taking the mean pixel intensity for each pixel. Thus, the highest diagnosticity pixels for an object are those which were marked most consistently as distinguishing it from the most distractors.

### Reporting summary

Further information on research design is available in the Nature Portfolio Reporting Summary linked to this article.

## Supplementary information

Supplementary Information

Reporting Summary

## Data Availability

All data for results presented in this article is available in the following GitHub repository: https://github.com/hawkrobe/graphical_conventions.

## Code availability

All code for results presented in this article is available in the following GitHub repository: https://github.com/hawkrobe/graphical_conventions.
