# Supplementary material for: Visual resemblance and interaction history jointly constrain pictorial meaning
Source: Nat Commun. 2023 Apr 17;14:2199. doi: 10.1038/s41467-023-37737-w (PMC10110538; doi:10.1038/s41467-023-37737-w)
Supplement: Supplementary file 1 — Supplementary Information [file 41467_2023_37737_MOESM1_ESM.pdf]

Visual resemblance and interaction history jointly constrain pictorial meaning (Supplementary Materials)

Robert D. Hawkins[1,2*], Megumi Sano[1], Noah D. Goodman[1,3] and Judith E. Fan[1,4*]

[1]Department of Psychology, Stanford University.

[2]Department of Psychology, Princeton University.

[3]Department of Computer Science, Stanford University.

[4]Department of Psychology, University of California, San Diego.

*Corresponding author(s). E-mail(s): rdhawkins@princeton.edu; jefan@stanford.edu;

Supplementary Figures

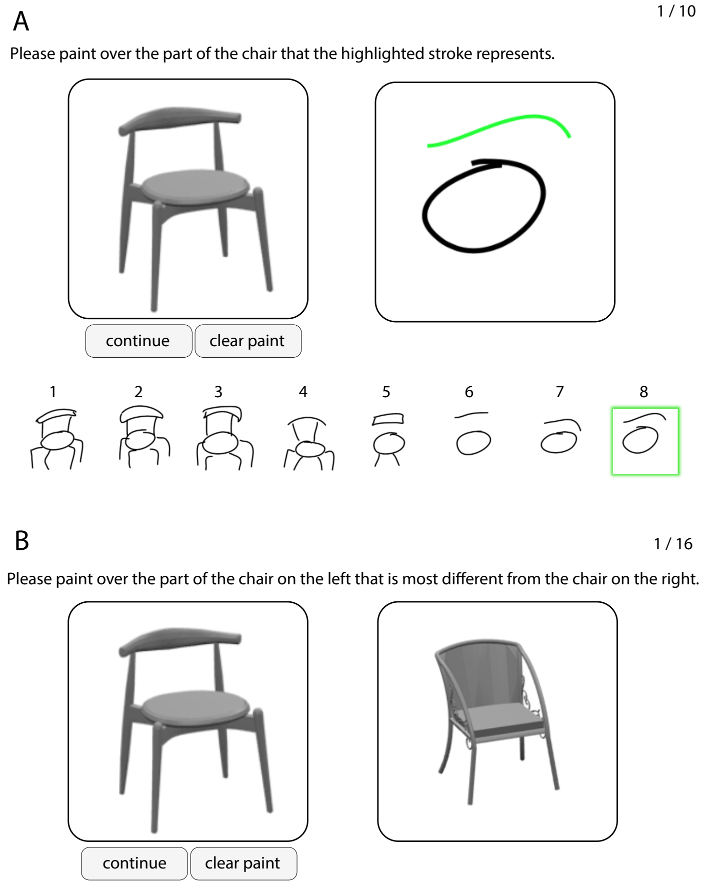

Supplementary Figure 1: **Interfaces for sketch-to-object mapping and diagnosticity mapping tasks.** (A) Task interface provided to annotators who indicated which parts of the object each stroke of each drawing corresponded to. (B) Task interface provided to annotators who indicated which part of a target object (left) was most different from the distractor object (right). These annotations were obtained for all pairs of objects from each context, which were then aggregated to produce a graded diagnosticity map for each object. Images of objects were rendered from 3D mesh models in the ShapeNet database and appear in this figure with permission.

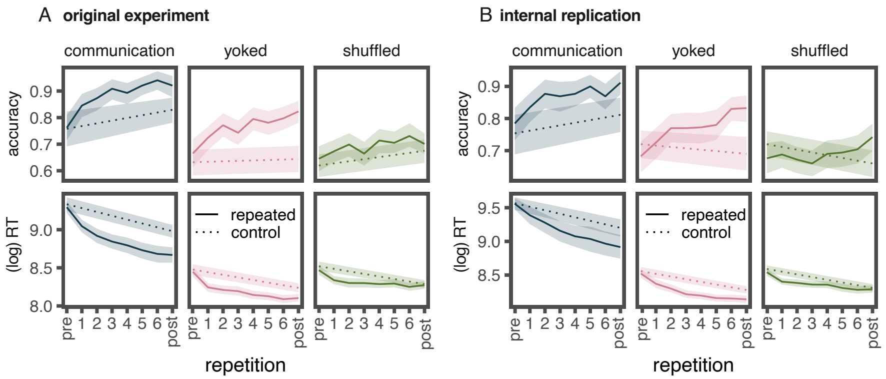

Supplementary Figure 2: **Performance in accuracy and response time.** For both (A) the original experiment and (B) our internal replication, we decomposed our composite efficiency metric into raw accuracy (top row) and raw (logged) response times in milliseconds (bottom row). We observe that practice effects for naive scorers in the recognition experiments (measured by performance changes on control objects; dotted line) were weaker or comparable to practice effects observed in the original reference game.

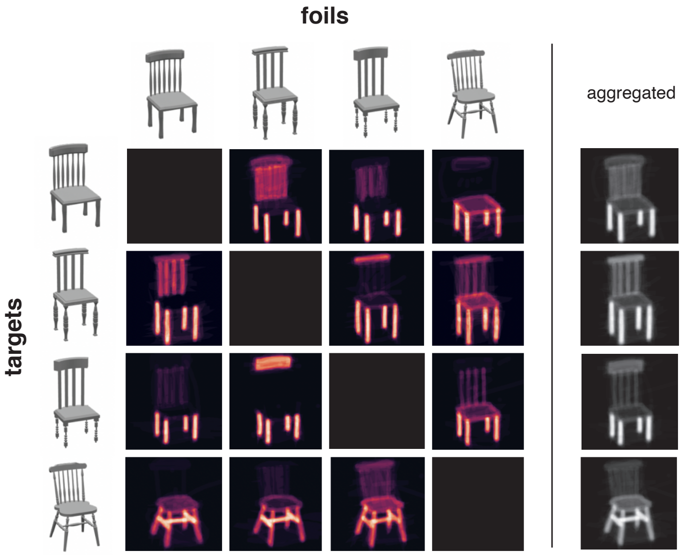

Supplementary Figure 3: **Pipeline for obtaining aggregate diagnosticity maps from pairwise annotations.** Maps for each target object (rows) were constructed by combining the raw diagnosticity maps (columns) obtained from pairing the target object with each of the three distractor objects. Different regions of the target object were diagnostic for each distractor; the aggregated map captures those regions which were identified by annotators, on average, across all distractors. Images of objects were rendered from 3D mesh models in the ShapeNet database and appear in this figure with permission.

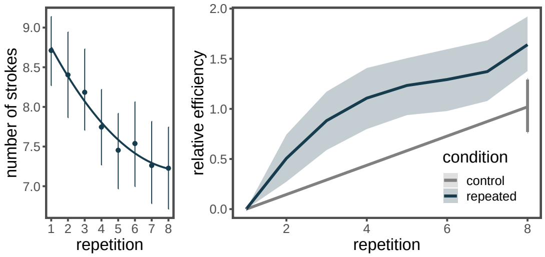

Supplementary Figure 4: **Selected results from internal replication.** *Left*: The number of strokes used to produce drawings across repetitions. *Right:* Communication efficiency increases across repetitions. Efficiency combines both speed and accuracy, and is plotted relative to the first repetition. Error ribbons and bars represent 95% CI.

Supplementary Methods

Our diagnosticity analyses (Section 2.4) required a larger sample size within each specific context, motivating a full replication of our study. In addition to providing data that is uniquely suited for measuring diagnosticity, this replication also provided an opportunity to internally validate our results from earlier sections in an independent sample. In this section, we report our findings using the same analysis pipeline on these new data ($N = 66$ dyads; see Supplementary Figure S4). Unless otherwise stated, we used exactly the same mixed-effects model structure on both datasets.

A2.1: Replicating improvements in communicative efficiency

Efficiency

In Section 2.1 of the main text, we modeled the change in efficiency (BIS scores) in the repeated condition using a mixed-effects model with linear and quadratic fixed effects of repetition block along with random effects of repetition block for each dyad (intercepts are unnecessary as they have already been z-scored):

```
BIS ~ poly(repetition, 2) + (0 + poly(repetition, 1) |
gameID)
```

Here we fit the same models to the communication experiment data from the internal replication. We again computed the balanced integration score (BIS) and found a significant improvement in communicative efficiency in the repeated condition, $b = 21.5, t = 12.1, p < 0.001$, similar to what we had estimated in the original communication experiment ($b = 23.5, t = 13.5$).

Accuracy and response time

We also conducted the same secondary analyses of drawing time and accuracy (using a logistic linking function to model binary outcomes.)

```
correct ~ poly(repetition, 2) + (1 + poly(repetition, 1) |
gameID)
```

```
RT ~ poly(repetition, 2) + (1 + poly(repetition, 1) | gameID)
```

We replicated our initial findings that drawing time decreased across repetitions, $b = -77.2, t(65) = -9.4, p < 0.001$, and accuracy increased across repetitions, $b = 16.8, z = 4.4, p < 0.001$ (see Supplementary Figure S2B). These parameter estimates were comparable to those we had obtained in the original communication experiment: $b = -69.9, t(66) = -11.5$ and $b = 26.6, z = 5.8$, respectively.

Number of strokes

Finally, we replicated our analyses of how the number of strokes produced in each drawing changed across repetitions.

```
numStrokes ~ poly(repetition, 2) + (1 + poly(repetition, 1) |
gameID)
```

This analysis revealed that the total number of strokes comprising each drawing consistently decreased, $b = -23.4, t(65) = -4.9, p < 0.001$ (similar to what we had found in the original communication experiment, $b = -22.9, t = -6.00$). Because a modification in the replication design prevented viewers from interrupting (unlike the original experiment when a viewer could make a response at any point), this result represents a purer measure of how many strokes the sketcher independently *decided* to keep adding, implying that these changes were not solely driven by the viewer's interruptions.

Bayesian regressions

Our analyses treated repetition number as a continuous (integer) predictor, including both linear and quadratic effects (to allow for non-linearities). However, these assumptions may be too strong; additionally, in some cases the full random-effects structure led to singularities during the fitting procedure. To check the robustness of our findings, we fit the corresponding Bayesian mixed-effects regression models with the weaker assumption that the effect of repetition is monotonic [1]. We included the full random effects structure (intercepts and monotonic effects of

repetition for each dyad). We found reliable improvements in performance across successive repetitions for all metrics, including efficiency ($b = 0.26$, 95% CI= $[0.23, 0.29]$ for original data; $b = 0.23$; 95% CI= $[0.19, 0.26]$ for replication), raw accuracy (Bernoulli linking function; $b = 0.28$; 95% CI= $[0.18, 0.39]$ for original data; $b = 0.19$; 95% CI= $[0.10, 0.30]$ for replication), drawing time ($b = -0.74$; 95% credible interval= $[-0.87, -0.61]$ for original data; $b = -0.76$; 95% CI= $[-0.93, -0.58]$ for replication), and number of strokes ($b = -0.24$; 95% credible interval= $[-0.32, -0.16]$ for original data; $b = -0.21$; 95% CI= $[-0.30, -0.12]$ for replication).

A2.2: Improvements in communication are object-specific

Efficiency

In Section 2.2, we compared the change in communication efficiency across phases (pre vs. post phases) for each condition (repeated vs. control objects). Both categorical predictors were effect-coded for interpretability of main effects:

```
BIS ~ phase * condition + (0 + phase * condition | gameID)
```

In our internal replication, we found a similar overall main effect of performance improvement between the pre and post phases for all objects, $b = 0.66$, $t(67) = 11.1$, $p < 0.001$ (compared to the effect in the original communication experiment, $b = 0.72$, $t(137) = 14.6$), as well as the predicted interaction, $b = -0.16$, $t = -3.7$, $p < 0.001$ (compared to the effect in the original communication experiment, $b = -0.16$, $t = -3.1$). A Bayesian mixed-effects regression model with full random effect structure at the dyad level yielded a similar effects of phase, $b = 0.67$, 95% CI = $[0.55, 0.78]$; (compared to $b = 0.72$, 95% CI = $[0.62, 0.83]$ in the original communication experiment) and interaction coefficient estimate, $b = -0.16$, 95% CI = $[-0.24, -0.07]$ (compared to $b = -0.16$, 95% CI= $[-0.27, -0.06]$ in the original communication experiment).

Accuracy and response time

Next we analyzed raw accuracy and drawing time separately:

```
correct ~ phase * condition + (1 + phase * condition | gameID)
```

```
RT ~ phase * condition + (1 + phase * condition | gameID)
```

For the pure accuracy model, we found a marginal interaction in the replication (control: +5.8%, repeated: +12.7%, $b = -0.25, z = -1.85, p = 0.065$), which is statistically weaker than our original effect (control: +7.1%, repeated: +14.5%, $b = -0.46, z = -2.8$). For the pure drawing time model, we found a significant interaction, $b = 0.41, t(465) = 3.77, p < 0.001$) (compared to $b = 0.37, t(113) = 2.8, p = 0.006$). The corresponding Bayesian mixed-effects regression models with full random effect structure at the dyad level yielded comparable interaction coefficients for raw accuracy, $b = -0.20$, 95% CI = $[-0.40, -0.00]$; (compared to $b = -0.26$, 95% credible interval = $[-0.51, -0.05]$ in the original data) and raw response time, $b = 0.42$, 95% CI = $[0.20, 0.64]$ (compared to $b = 0.38$, 95% CI = $[0.11, 0.64]$).

Convergence within dyad

Finally, we again extracted high-dimensional visual feature vectors using the penultimate layer of the ConvNet VGG-19 (i.e., `fc6`)to analyze how the internal consistency between successive drawings changed over time:

```
sim-to-previous ~ poly(rep, 2) + (1 + poly(rep, 1) || gameID) + (1 + poly(rep, 1) || target)
```

We found that successive drawings of the same object made by the same sketcher became more similar over time, $b = 0.57, t(7.6) = 4.2, p = 0.003$, consistent with our original findings ($b = 0.62, t(12) = 3.84$). Similar effects were found using a Bayesian mixed-effects regression model with monotonic predictors, $b = 0.01$; 95% CI= $[0.009, 0.013]$, consistent with original findings ($b = 0.01$, 95% CI= $[0.006, 0.012]$).

A2.3: Performance gains depend on shared interaction history

Efficiency

We conducted a replication of our control experiment using the new drawings we collected in our replication of the reference game. For this control experiment, we recruited 100 naive viewers for the ‘yoked’ condition and 125 naive viewers for the ‘shuffled’ condition. We fit the same linear mixed-effects model, controlling for non-linearities with a quadratic term:

```
bis ~ version * poly(repetition, 2) + (1 + version *
poly(repetition, 1) || orig_gameID)
```

As before, we found a significant main effect of repetition on recognition performance across both ’yoked’ and ’shuffled’ condition, $b = 40.3, t = 15.1, p < 0.001$ (compared to our original effect of $b = 34.8, t = 12.4$), as well as a significant interaction, $b = -18.6, t = -3.9, p < 0.001$ (compared to our original effect of $b = -16.9, t = -4.9$), indicating that the benefits of repetition again accrued for the ‘shuffled’ group to a lesser extent than the ‘yoked’ group. Although it was not our primary comparison of interest, we found no significant interaction for the ‘yoked’ vs. original ‘communication’ group, $b = -2.5, t = -0.8, p = 0.4$ (compared to our original effect of $b = -8.4, t = -2.6, p = 0.008$). It is possible that presenting completed static drawings in the follow-up communication experiment, rather than stroke-by-stroke as in the original communication experiment, brought these conditions closer together.

Divergence across dyads

Finally, we examined the extent to which drawings diverge across interactions by analyzing high-dimensional visual features. We found that drawings of the same object produced in different interactions became significantly less similar to each other over time, $b = -2.0, t = -4.99, p = 0.001$, consistent with our original result ($b = -1.4, t = -2.5$).

Supplementary Note 1

Model-based analyses of object-specific information in drawings

The model-based feature analyses reported in our manuscript focus on making comparisons between drawings of the same object. The validity of these analyses relies on the assumption that there is fine-grained information about specific objects available in these model features. To evaluate this assumption, we conducted an additional analysis to measure the degree to which drawings of one object are distinguishable from drawings of another object using these model-based features. Here we used a classifier-based approach to measure this similarity: insofar as drawings of the same object are sufficiently distinct from drawings of another object, then a linear classifier trained to distinguish drawings of different objects should achieve above-chance accuracy. Specifically, we fit and evaluated a logistic-regression classifier trained with L2-regularization under 5-fold cross-validation on all drawings from the pre phase (i.e., before participants had the opportunity to develop object-specific, interaction-specific conventions). This classifier achieved 32.7% accuracy (standard deviation across splits: 6.08%), which was well above that expected by chance (6.25%), providing robust evidence for object-specific information in these drawings using these model-based feature representations.

Another assumption we make when interpreting our finding that successive drawings of the same object produced by the same sketcher participant increase in similarity is that these changes are due to factors other than an increasing amount of empty space on the canvas across repetitions, which would also contribute to an increase in visual similarity. To test this alternative account, we analyzed the degree to which successive drawings of the same object were more similar to one another than successive drawings of different objects, all produced by the same participant. We found indeed that successive drawings of the same object were reliably more similar to one another than successive drawings of different objects (difference in correlation distance: $\Delta r = -0.13$; $95\% CI : [-0.14, -0.11]$). These findings argue against the

notion that the increase in similarity between successive drawings of the same object is primarily driven by an increasing amount of empty space on the drawing canvas.

Supplementary References

[1] Bürkner, P.-C. & Charpentier, E. Modelling monotonic effects of ordinal predictors in bayesian regression models. *British Journal of Mathematical and Statistical Psychology* **73** (3), 420–451 (2020) .
