# Supplementary material for: Visual resemblance and interaction history jointly constrain pictorial meaning
Source: Nat Commun. 2023 Apr 17;14:2199. doi: 10.1038/s41467-023-37737-w (PMC10110538; doi:10.1038/s41467-023-37737-w)
Supplement: Supplementary file 2 — Reporting Summary [file 41467_2023_37737_MOESM2_ESM.pdf]

nature portfolio

Corresponding author(s): Judith Fan

Last updated by author(s): Nov 14, 2022



# Reporting Summary

Nature Portfolio wishes to improve the reproducibility of the work that we publish. This form provides structure for consistency and transparency in reporting. For further information on Nature Portfolio policies, see our Editorial Policies and the Editorial Policy Checklist.

## Statistics

For all statistical analyses, confirm that the following items are present in the figure legend, table legend, main text, or Methods section.

| n/a | Confirmed | |
|---|---|---|
| ☐ | ☒ | The exact sample size (*n*) for each experimental group/condition, given as a discrete number and unit of measurement |
| ☐ | ☒ | A statement on whether measurements were taken from distinct samples or whether the same sample was measured repeatedly |
| ☐ | ☒ | The statistical test(s) used AND whether they are one- or two-sided<br>*Only common tests should be described solely by name; describe more complex techniques in the Methods section.* |
| ☐ | ☒ | A description of all covariates tested |
| ☒ | ☐ | A description of any assumptions or corrections, such as tests of normality and adjustment for multiple comparisons |
| ☐ | ☒ | A full description of the statistical parameters including central tendency (e.g. means) or other basic estimates (e.g. regression coefficient) AND variation (e.g. standard deviation) or associated estimates of uncertainty (e.g. confidence intervals) |
| ☐ | ☒ | For null hypothesis testing, the test statistic (e.g. *F*, *t*, *r*) with confidence intervals, effect sizes, degrees of freedom and *P* value noted<br>*Give P values as exact values whenever suitable.* |
| ☒ | ☐ | For Bayesian analysis, information on the choice of priors and Markov chain Monte Carlo settings |
| ☐ | ☒ | For hierarchical and complex designs, identification of the appropriate level for tests and full reporting of outcomes |
| ☒ | ☐ | Estimates of effect sizes (e.g. Cohen's *d*, Pearson's *r*), indicating how they were calculated |

*Our web collection on statistics for biologists contains articles on many of the points above.*

## Software and code

Policy information about availability of computer code

| | |
|---|---|
| Data collection | Our data were collected over the web, and all code used to run our experiments are available in the `experiments` directory of our github repository: https://github.com/hawkrobe/graphical_conventions |
| Data analysis | All analyses of our behavioral data, including pre-processing steps, are available in the `analysis` directory of our github repository; https://github.com/hawkrobe/graphical_conventions |

For manuscripts utilizing custom algorithms or software that are central to the research but not yet described in published literature, software must be made available to editors and reviewers. We strongly encourage code deposition in a community repository (e.g. GitHub). See the Nature Portfolio guidelines for submitting code & software for further information.

## Data

Policy information about availability of data

All manuscripts must include a data availability statement. This statement should provide the following information, where applicable:

- Accession codes, unique identifiers, or web links for publicly available datasets
- A description of any restrictions on data availability
- For clinical datasets or third party data, please ensure that the statement adheres to our policy

All raw data collected for this manuscript has been released publicly in the `data` directory of our github repository: https://github.com/hawkrobe/graphical_conventions

# Field-specific reporting

Please select the one below that is the best fit for your research. If you are not sure, read the appropriate sections before making your selection.

☐ Life sciences ☒ Behavioural & social sciences ☐ Ecological, evolutionary & environmental sciences

For a reference copy of the document with all sections, see nature.com/documents/nr-reporting-summary-flat.pdf

# Behavioural & social sciences study design

All studies must disclose on these points even when the disclosure is negative.

| | |
|---|---|
| Study description | All of the studies we report are fully randomized quantitative behavioral experiments. |
| Research sample | We recruited participants on Amazon Mechanical Turk, a popular crowdsourcing platform. While not fully representative of the adult population in the United States, the demographics of this platform are well-documented to be more representative than undergraduate students (e.g. by Difallah, Filatova, & Ipeirotis, 2018; https://doi.org/10.1145/3159652.3159661) |
| Sampling strategy | A convenience sample was recruited via Amazon Mechanical Turk; the final sample size was determined by recruiting twice as many participants as we expected based on a power analysis conducted on a pilot dataset. After performing all planned analyses, we additionally conducted a full internal replication with the same sample size as the original study. |
| Data collection | Data was collected directly in participants' web browsers. |
| Timing | Our first round of data collection continued from the July 2018 through December 2018. We completed our internal replication in January 2019. Finally, we collected annotations in June 2020. |
| Data exclusions | We excluded communication-game data from 5 participants who did not meet our prespecified inclusion criteria. We excluded recognition data from 22 participants who did not meet our pre-specified inclusion criteria for accurate and consistent response on attention-check trials. |
| Non-participation | No participants declined participation. In 11 sessions during the communication experiment, however, the connection between participants and our experiment server was interrupted, leading those sessions to terminate early and precluding analysis of their data. |
| Randomization | Participants were randomly allocated to dyads, though our primary experimental manipulations were conducted within-dyad. |

# Reporting for specific materials, systems and methods

We require information from authors about some types of materials, experimental systems and methods used in many studies. Here, indicate whether each material, system or method listed is relevant to your study. If you are not sure if a list item applies to your research, read the appropriate section before selecting a response.

## Materials & experimental systems

| n/a | Involved in the study | |
|---|---|---|
| ☒ | ☐ | Antibodies |
| ☒ | ☐ | Eukaryotic cell lines |
| ☒ | ☐ | Palaeontology and archaeology |
| ☒ | ☐ | Animals and other organisms |
| ☐ | ☒ | Human research participants |
| ☒ | ☐ | Clinical data |
| ☒ | ☐ | Dual use research of concern |

## Methods

| n/a | Involved in the study | |
|---|---|---|
| ☒ | ☐ | ChIP-seq |
| ☒ | ☐ | Flow cytometry |
| ☒ | ☐ | MRI-based neuroimaging |

## Human research participants

Policy information about studies involving human research participants

| | |
|---|---|
| Population characteristics | See above. |
| Recruitment | See above. |
| Ethics oversight | Participants provided informed consent in accordance with the Stanford University IRB. |

Note that full information on the approval of the study protocol must also be provided in the manuscript.
